# The Biological and Prognostic Implications of the Nicotinic Acetylcholine Receptor *α*3, *α*5, and *α*7 Subunits in Oral Squamous Cell Carcinoma

**DOI:** 10.1002/cam4.71358

**Published:** 2025-11-07

**Authors:** Chi‐Maw Lin, Long‐Wei Lin, Tseng‐Cheng Chen, Yi‐Ling Ye, Bor‐Luen Chiang

**Affiliations:** ^1^ Department of Otolaryngology National Taiwan University Hospital, Yun‐Lin Branch Yun‐Lin Taiwan; ^2^ Graduate Institute of Clinical Medicine, College of Medicine National Taiwan University Taipei Taiwan; ^3^ Department of Pathology National Taiwan University Hospital, Yun‐Lin Branch Yun‐Lin Taiwan; ^4^ Department of Otolaryngology National Taiwan University Hospital and National Taiwan University, College of Medicine Taipei Taiwan; ^5^ Department of Biotechnology National Formosa University Yun‐Lin Taiwan

**Keywords:** antigen presentation, cell adhesion, cholinergic receptor nicotinic alpha, epithelial–mesenchymal transition, immune reactions, nicotinic acetylcholine receptor

## Abstract

**Background:**

The divergent loop structures of nicotinic acetylcholine receptor (nAChR) *α*3, *α*5, and *α*7 subunits (encoded by *CHRNA3*, *CHRNA5*, and *CHRNA7*) are involved in kinase phosphorylation and signal transduction, potentially affecting oral squamous cell carcinoma (OSCC), the most common head and neck cancer (HNC). However, their specific roles in OSCC remain unclear.

**Methods:**

We integrated analyses of SCC‐4 tongue cancer cells with *CHRNA* overexpression, immunohistochemistry of OSCC pathological specimens, and data from the cancer genome atlas (TCGA), DepMap, and Puram 2017 to assess *CHRNA3*, *CHRNA5*, and *CHRNA7* in OSCC/HNC.

**Results:**

In OSCC, *CHRNA3*, *CHRNA5*, and *CHRNA7* expression interacted with epithelial–mesenchymal transition (EMT) markers and correlated with invasive patterns. *CHRNA3* reduced epithelial and enhanced mesenchymal traits, supporting EMT. *CHRNA5* further promoted mesenchymal features, was linked to disseminated tumor patterns, and predicted poor prognosis. *CHRNA7* enhanced both epithelial and mesenchymal markers, maintaining a hybrid EMT state. In HNC, bioinformatic analyses revealed that *CHRNA3* preserved ion channel activity, *CHRNA5* promoted DNA replication, reduced adhesion, suppressed antigen presentation, and induced hypomethylation and miRNA overexpression, while *CHRNA7* promoted differentiation with variable effects on adhesion and antigen presentation. In DepMap HNC cell lines, high *CHRNA3*/*CHRNA5* and low *CHRNA7* expression were associated with resistance to most inhibitors. Epidermal growth factor receptor (EGFR) inhibitors were effective in *CHRNA3*/*CHRNA*7‐high HNC, whereas cyclin‐dependent kinase (CDK) inhibitors were effective in *CHRNA5*‐high HNC.

**Conclusion:**

Differential expression of *CHRNA3*, *CHRNA*5, and *CHRNA7* indicates different EMT states in OSCC/HNC, influencing proliferation, differentiation, cell adhesion, immune reactions, and treatment efficacy, and warrants further experimental validation.

## 1 Introduction

Smoking and its major ingredient nicotine are responsible for the development and deterioration of head and neck cancer (HNC), which accounted for more than 744,000 new cases and 364,000 deaths in 2020, the majority of which were oral squamous cell carcinoma (OSCC) [1, 2, 3, 4]. The nicotinic acetylcholine receptors (nAChRs), which are activated by exogenous nicotine in cigarettes or by endogenous acetylcholine (ACh) in cancer cells, are expressed on both neuronal and nonneuronal tissues and are involved in tobacco dependence as well as multiple tumorigenic signaling pathways, facilitating HNC growth and metastasis [5, 6]. The nAChRs are ligand‐gated ion channels that consist of five homomeric or heteromeric subunits (*α*, *β*, γ, ε, and δ) with different isoforms of the *α* and *β* subunits (*α*1‐*α*10, *β*1‐*β*4) [5, 6]. The *α* subunit, which is encoded by the *CHRNA* gene, harbors a disulfide bond that is essential for the ligand‐binding orthosteric site, and a functional nAChR requires at least two *α* subunits [5, 7].

In our previous work, we explored the expression of different nAChR subunits in OSCC and found that the *α*3, *α*5, and *α*7 subunits were related to unchanged, decreased, and increased epithelial features, respectively [8]. An increase in subunit *α*5 was also associated with a decrease in local immune cells and a worse prognosis, while increases in subunit *α*3 and *α*7 were related to increased local immune cells in OSCC [8]. The *α*7 subunits commonly form the homomeric nAChR, whereas the *α*3 and *α*5 subunits could only join in the heteromeric nAChR (Figure S1) [6, 9]. Subunit *α*5 is generally thought to assemble only in the accessory subunit of the nAChR (there was at most one accessory subunit among a total of five subunits of the nAChR), which did not directly participate in the formation of orthosteric binding sites [9]. The main polypeptide chains of subunit *α*3, *α*5, and *α*7 are highly similar but the large cytoplasmic M3–M4 loops of them differ greatly in the AlphaFold protein structure database (Figure S1) [10, 11, 12]. This loop contains the phosphorylation sites for various tyrosine and serine/threonine kinases, which are involved in downstream nonionic signal transduction [6, 9]. The divergent loop composition and structure of the *α*3, *α*5, and *α*7 subunits could influence tumor cells to exhibit different biological behaviors. Nevertheless, there is still limited knowledge about the effects of different nAChR subunits on OSCC/HNC. Therefore, we conducted this study using the SCC‐4 tongue cancer cell line, immunohistochemistry (IHC) of OSCC pathological specimens, tissue buck sequencing database (The Cancer Genome Atlas; TCGA), cell line sequencing database (DepMap), and single‐cell RNA sequencing (scRNA‐seq) database (Puram 2017) [13]. Further gene set enrichment analysis (GSEA) and protein–protein interaction (PPI) network functional enrichment analysis were also adopted to interpret the influences of *CHRNA3*, *CHRNA5*, and *CHRNA7* in HNC.

## 2 Materials and Methods

### 2.1 Cell Culture, Transfection, and Stimulants

The SCC‐4 tongue cancer cell line was purchased from the Bioresource Collection and Research Center in Taiwan and cultured in Dulbecco's modified Eagle's medium/Ham's F12 (Gibco) supplemented with 10% fetal bovine serum (Gibco), 1% penicillin–streptomycin–amphotericin B (Gibco), and 400 ng/mL hydrocortisone (TargetMol). The cells were maintained at 37°C in a humidified 5% CO_2_ incubator.

Before transfection, the SCC‐4 cells were seeded in six‐well plates at a density of 2.5 × 10^5^ cells/2 mL and cultured for 24 h. Plasmid DNA expression vectors targeting *CHRNA3*, *CHRNA5*, *CHRNA7*, *DSG2*, and *TGFBR2* (costum synthesis by GeneDireX) (2.5 μg of each expression vector) were successively transfected into the SCC‐4 cells using Lipofectamine 2000. Nicotine (1 μM) (TargetMol) and TGF*β*1 (2.5 ng/mL) (Gibco) were used as stimulants for nAChRs and TGFBRs, respectively.

### 2.2 Quantitative Reverse Transcription Polymerase Chain Reaction (qRT‐PCR)

The transfected SCC‐4 cells were cultured for 48 h, and total RNA was extracted from the cells using a Total RNA Isolation Kit (GeneDireX). Afterward, total RNA was reverse‐transcribed to complementary DNA (cDNA) using a GScript First‐Strand Synthesis Kit (GeneDireX). Quantitative PCR of cDNA was subsequently performed with MorreSYBR qPCR Master Mix (MORREBIO) using paired primers for *CHRNA3*, *CHRNA5*, *CHRNA7*, *CDH1*, *EPCAM*, *ESRP1*, *ESRP2*, *KRT5*, *KRT14*, *DSG2*, *ACTA2*, *VIM*, *TGFBR2*, and *GAPDH* (designed by GeneDireX). *GAPDH* was used as the endogenous control. The normalized fold change in the expression of each gene between different groups was calculated using the 2^−∆∆Ct^ method based on the gene with the highest Ct value.

### 2.3 RNA Extraction and RNA Sequencing (RNA‐Seq)

The seeded SCC‐4 cells were collected after 48 h of incubation, and their total RNA was extracted by Trizol (Invitrogen). The RNA concentration was determined by the NanoDrop One spectrophotometer (Thermo Fisher), and the RNA integrity was assessed by the 2100 Bioanalyzer instrument (Agilent). Total RNA was sent for library preparation utilizing the Illumina Stranded mRNA Prep, Ligation kit, and RNA‐seq was performed with paired‐end 150 base pairs by Illumina NovaSeq 6000.

The processes of upstream data analyses included the adapter trimming (BBDuk), the quality control (FastQC), and the quantification of gene expression (Salmon) [14]. The downstream data analyses were performed on the platform of ExpressAnalyst, in which the differentially expressed genes (DEGs) were explored using the DESeq2 method [15, 16].

### 2.4 Patient Collections and IHC Staining

OSCC patients diagnosed during 2014–2016 at National Taiwan University Hospital, Yun‐Lin Branch were retrospectively enrolled, and their clinical follow‐up data were recorded up to July 2021. The ICD‐10 codes for these OSCC patients are C00, C02–C06, and C14. Patients lacking pathological reports or lost to follow‐up after treatment were excluded. The TNM (tumor, node, and metastases) status of OSCC was classified according to the 2018 criteria of the American Joint Committee on Cancer (AJCC) [17].

The IHC staining of nAChR *α*3, *α*5, and *α*7 subunits was performed using formalin‐fixed, paraffin‐embedded (FFPE) primary tumor sections according to a standard protocol [8]. The antibody–antigen complex was visualized using diaminobenzidine tetrachloride (DAB) as the chromogen. Primary antibodies specific to the nAChR *α*3, *α*5, and *α*7 subunits were obtained from Abcam (ab183097), Thermo Fisher Scientific (PA5‐79046), and Abcam (ab216485), respectively.

Images of the stained slides were captured at 200× magnification. The expression levels of nAChR *α*3, *α*5, and *α*7 subunits were quantified using ImageJ (version 1.53 k) with the plugin IHC‐toolbox [18]. The detection threshold was set at 170 for the 8‐bit grayscale images, and the percentage of stained areas in the entire image was used to represent IHC expression levels.

### 2.5 Database Processing

Clinical and bulk RNA‐seq data from the TCGA OSCC and HNC cohorts were assessed via cBioPortal (Firehose Legacy) and UCSC Xena (GDC TCGA Head and Neck Cancer) [19, 20, 21, 22]. RNA‐seq and drug sensitivity data for HNC cell lines were retrieved from DepMap [23, 24]. scRNA‐seq data of the HNC cohort (Puram 2017) were acquired from UCSC Xena [13]. The functional enrichment analysis of PPI networks was implemented using the STRING database [25]. The Gene Ontology (GO) term was adopted for pathway enrichment analysis [26, 27, 28, 29, 30, 31].

### 2.6 Statistical Analyses

The statistical calculations and graphical delineations of the comparative studies were executed using GraphPad Prism (version 9), Stata (version 13), and R (version 4.2.2). For RNA‐seq data, normalized counts were utilized in the differential expression gene (DEG) analyses. Fragments per kilobase of transcript per million mapped reads (FPKM) or transcripts per million (TPM) were log_2_(FPKM + 1) or log_2_(TPM + 1) transformed and utilized in the comparison of specific gene expression levels. DEG analysis was performed using the ExpressAnalyst platform (for our RNA‐seq data), the cBioPortal platform (for TCGA), the DESeq2 package in R (for DepMap), and the multiple *T* tests (for Puram 2017 because normalized counts were unavailable). GSEA was performed using the clusterProfiler package in R [32]. Donut charts, pie charts, forest plots, and Venn diagrams were created using Excel (Microsoft 365), PowerPoint (Microsoft 365), the forestploter package in R, and an online tool (https://bioinformatics.psb.ugent.be/webtools/Venn/), respectively. A q value or adjusted *p* value less than 0.05 was considered statistically significant in the multiple T tests, DEGs, and enrichment analyses. Otherwise, a *p* value below 0.05 indicated a statistically significant result.

## 3 Results

### 3.1 *CHRNA3* , *CHRNA5* , *CHRNA7* , as well as Epithelial and Mesenchymal Markers Mutually Influenced Each Other in SCC‐4 Cells

**FIGURE 1:**
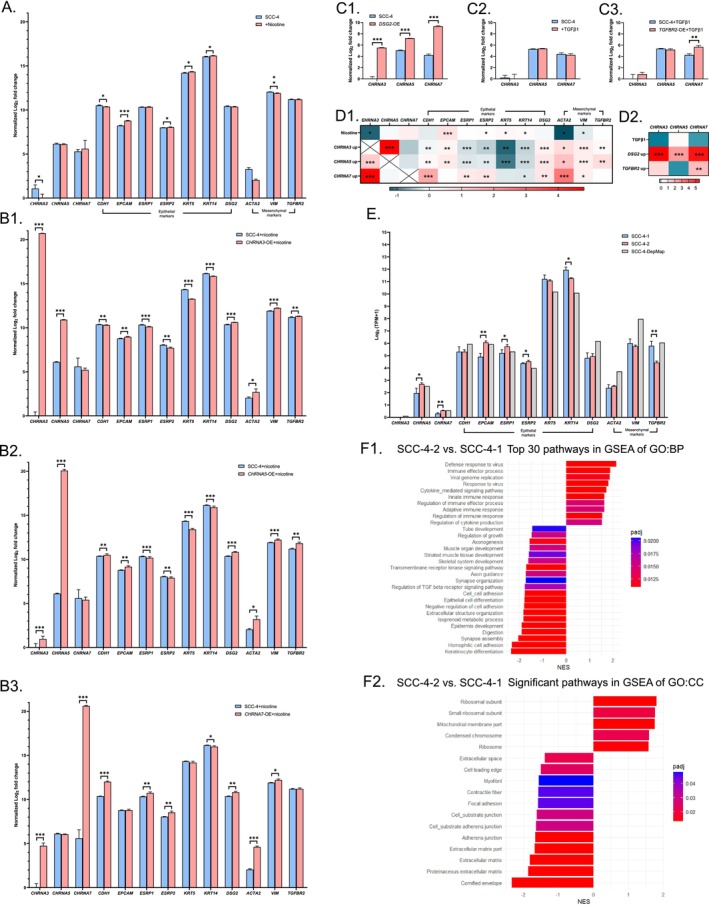
Influences of *CHRNA3*, *CHRNA5*, and *CHRNA7* expression in SCC‐4 tongue cancer cells. (A) The effects of nicotine (1 μM) treatment (qRT‐PCR). (B1–B3) The effects of overexpressed *CHRNA3*, *CHRNA5*, and *CHRNA7*, respectively (qRT‐PCR). (C1–C3) The effects of overexpressed *DSG2*, TGF*β*1 treatment (2.5 ng/mL), and overexpressed *TGFBR2*, respectively (qRT‐PCR). (D1–D2) Summary of the qRT‐PCR results. (E) The expression patterns of our SCC‐4 cells at two different time points (SCC‐4‐1 and SCC‐4‐2) and the DepMap SCC‐4 cells (RNA‐seq). (F1–F2) GSEA results comparing SCC‐4‐2 cells to SCC‐4‐1 cells. qRT‐PCR, quantitative reverse transcription polymerase chain reaction. RNA‐seq, RNA sequencing. TPM, transcripts per million. GO, gene ontology. BP, biological process. CC, cellular component. *, *p* < 0.05. **, *p* < 0.01. ***, *p* < 0.001.

The qRT‐PCR analysis revealed that *CHRNA5* expression was highest, followed by *CHRNA7* and then *CHRNA3*, in SCC‐4 tongue cancer cells (Figure 1A). Nicotine (1 μM) increased the expression of epithelial markers (*EPCAM*, *ESRP2*, and *KRT5/14*) but decreased the levels of *CHRNA3* and mesenchymal markers (*ACTA2* and *VIM*) (Figure 1A,D1). Overexpression of *CHRNA3*, *CHRNA5*, and *CHRNA7* via plasmid‐mediated transient transfection significantly increased the levels of *CHRNA5*, *CHRNA3*, and *CHRNA3*, respectively (Figure 1B,D1). Overexpression of *CHRNA3* and *CHRNA5* both increased the levels of all mesenchymal markers and decreased that of most epithelial markers, while overexpression of *CHRNA7* increased the expression of most epithelial and mesenchymal markers. On the other hand, overexpression of *DSG2* increased the expression of *CHRNA3*, *CHRNA5*, and *CHRNA7* (Figure 1,C1,D2). Although TGF*β*1 (2.5 ng/mL) did not significantly alter the expression of *CHRNA3*, *CHRA5*, or *CHRNA7*, overexpression of *TGFBR2* significantly increased the expression of *CHRNA7* (Figure 1,C2,C3,D2). These findings suggested that *CHRNA3*, *CHRNA5*, *CHRNA7*, as well as epithelial and mesenchymal markers mutually influenced each other. The effects of *CHRNA3* and *CHRNA5* on epithelial markers were similar but differed from those of *CHRNA7*. *CHRNA3* and *CHRNA5* appeared to promote the epithelial–mesenchymal transition (EMT) process, while *CHRNA7* likely facilitated the maintenance of a hybrid EMT state.

### 3.2 The Expression of *CHRNA5* and *CHRNA7* Were Related to Cell Adhesion and Immune Responses in SCC‐4 Cells

SCC‐4 cells used in our experiments were collected at two different time points (SCC‐4‐1 and SCC‐4‐2) and subjected to RNA‐seq analysis. Our SCC‐4 cells and the SCC‐4 cells in the DepMap database shared similarities in the expression patterns of *CHRNA3*, *CHRNA5*, *CHRNA7*, as well as epithelial and mesenchymal markers, which were compatible with our qRT‐PCR results (Figure 1E). The bench effects on gene expression in our SCC‐4 cells were further analyzed, and revealed that SCC‐4‐2 cells had higher expression of *CHRNA5*, *CHRNA7*, *EPCAM*, and *ESRP1/2*, and lower expression of *KRT14* and *TGFBR2* than SCC‐4‐1 cells. The GSEA revealed that SCC‐4‐2 cells had increased immune responses and decreased cell adhesion/junction compared to SCC‐4‐1 cells (Figure 1F). These findings suggested that the expression of *CHRNA5* and *CHRNA7* might be related to cell adhesion and immune responses, which warrants further verification.

### 3.3 Clinical Characteristics of Patients in Our Series and in the TCGA Database

We analyzed three patient cohorts: 57 OSCC patients from our series (IHC_OSCC) and two cohorts from the TCGA database, including 500 HNC patients (TCGA_HNC) and 308 OSCC patients (TCGA_OSCC) (Table S1). Compared with the TCGA cohorts, our series had a lower mean age (59.9 vs. 61.08–61.8 years), a higher proportion of male patients (91.23% vs. 67.53%–73.4%), a lower proportion of advanced T‐stage cases (22.81% vs. 53.4%–53.9%), and fewer node‐positive cases (31.58% vs. 47.2%–48.05%). The cohorts had similar proportions of smokers (54.39% vs. 54.22%–61.4%), while our series had a lower proportion of alcohol users (31.58% vs. 52.6%–56%) and included 45.61% betel nut chewers (data not available in the TCGA database). These differences might contribute to variations in the analysis results.

### 3.4 High Expression of nAChR Subunits *α*5 and *α*7 in Tumor‐Infiltrating Areas, and Low Expression of *α*3, *α*5, and *α*7 in Tumor Islands, Were Associated With Worse Pathological Features and Poor Prognosis in OSCC Patients

**FIGURE 2:**
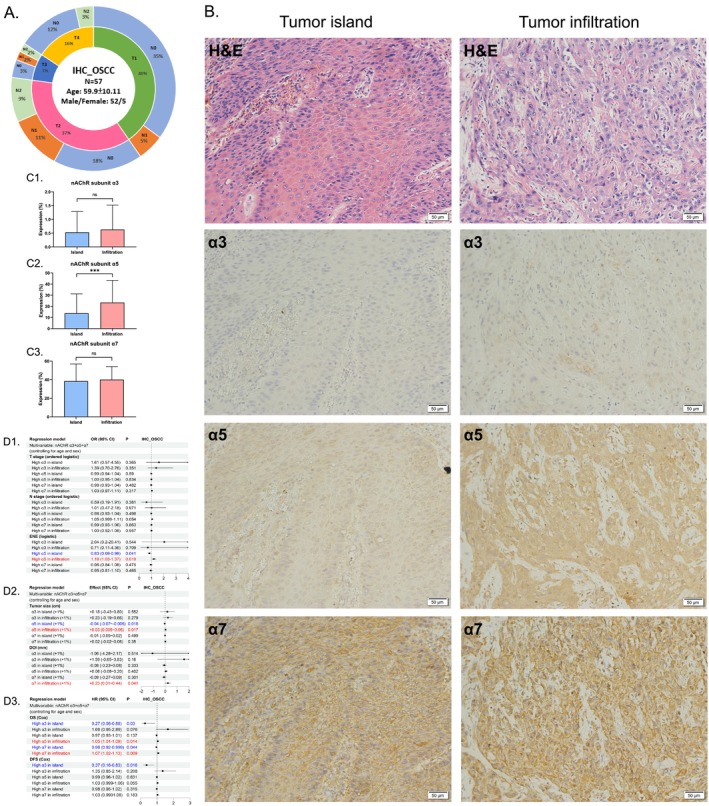
Influences of nAChR subunits *α*3, *α*5, and *α*7 expression in OSCC pathological specimens (IHC). (A) Clinical characteristics of the OSCC patients. (B) IHC staining of subunits *α*3, *α*5, and *α*7 in tumor islands and tumor infiltration. (C1–C3) Differences in the IHC levels of subunits *α*3, *α*5, and *α*7 in tumor islands and tumor infiltration, respectively. (D1–D3) The effects of subunits *α*3, *α*5, and *α*7 on pathological features and survival. nAChR, nicotinic acetylcholine receptor. IHC, immunohistochemistry; ENE, extranodal extension; DOI, depth of invasion; OS, overall survival; DFS, disease‐free survival; OR, odds ratio; HR, hazard ratio; CI, confidence interval. ***, *p* < 0.001. ns, nonsignificant.

Fifty‐seven OSCC patients were enrolled and the IHC staining of primary tumor specimens was performed. This cohort was male‐dominant, with a mean age of 59.9, and had the largest number of T1N0 and T2N0 patients (Figure 2A). The expression levels of nAChR *α*3, *α*5, and *α*7 subunits on the components of tumor islands (clusters of tumor cells with broad pushing margin) and tumor infiltration (clusters of tumor cells with finger‐like projection or diffuse invasion) were measured in the same section (Figure 2B). The tumor infiltration portion had a significantly higher subunit *α*5 level than the tumor island portion, whereas no significant difference was noted for subunit *α*3 or *α*7 (Figure 2C). Multivariable regression models (controlling for age and sex) revealed that a high subunit *α*3 level in tumor islands was related to better overall survival (OS) and disease‐free survival (DFS) (Figure 2D). A high subunit *α*5 level in tumor islands was related to smaller tumor size and a lower incidence of extranodal extension (ENE), while a high subunit *α*5 level in tumor infiltration was related to increased possibilities of ENE and worse OS. A high subunit *α*7 level in tumor islands was related to better OS, while a high subunit *α*7 level in tumor infiltration was associated with an increased depth of invasion (DOI) and worse OS. Regression models analyzing *α*3, *α*5, and *α*7 subunits individually in survival analysis, adjusted for age and sex, produced results generally consistent with those from a model including all three subunits simultaneously (Figure S2). These findings suggested that high expression of subunits *α*5 and *α*7 in tumor infiltration deteriorated the invasion patterns of OSCC and was related to a poor prognosis. Subunit *α*5 might facilitate a decrease in cell adhesion.

### 3.5 *CHRNA5* Had Greater Prognostic Impacts Than *CHRNA3* and *CHRNA7* in the TCGA_HNC and TCGA_OSCC Cohorts, and Was Related to Hypomethylation, miRNA Production, and a Decrease in Cell Adhesion and Immune Responses

**FIGURE 3:**
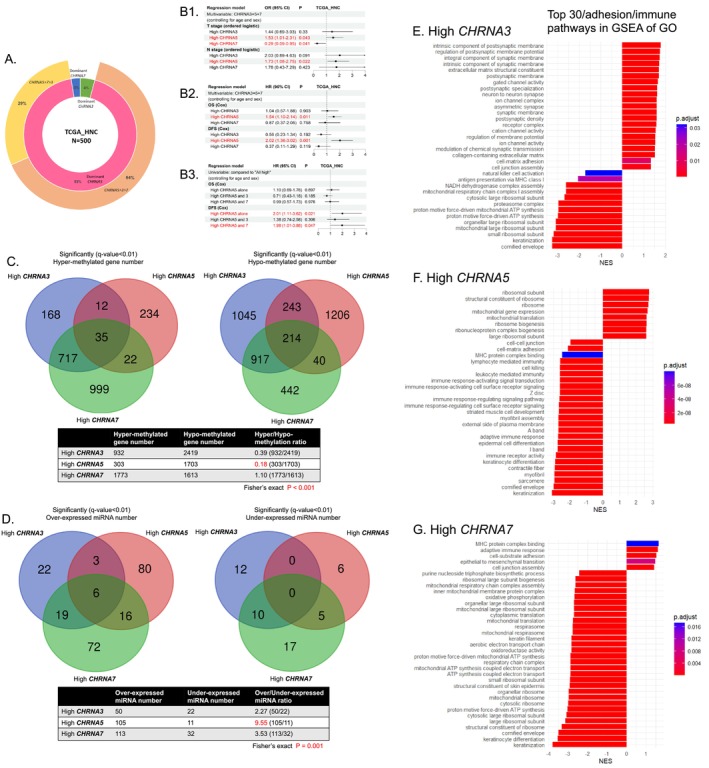
Influences of *CHRNA3*, *CHRNA5*, and *CHRNA7* expression in the TCGA‐HNC cohort. (A) Clinical characteristics of the TCGA‐HNC cohort. (B) The effects of *CHRNA3*, *CHRNA 5*, and *CHRNA7* expression on pathological features and survival. (C) The effects of *CHRNA3*, *CHRNA5*, and *CHRNA7* expression on DNA methylation. (D) The effects of *CHRNA3*, *CHRNA5*, and *CHRNA7* expression on miRNA production. (E–G) The enriched pathways in GSEA, GO for high *CHRNA3*, *CHRNA5*, and *CHRNA7* expression, respectively. HNC, head and neck cancer; OR, odds ratio; HR, hazard ratio; CI, confidence interval; GSEA, gene set enrichment analysis; GO, Gene ontology.

Two cohorts of 500 HNC and 308 OSCC patients were identified in the TCGA database, in which most of the primary tumors were *CHRNA5‐*dominant (93%–95%) (Bulk RNA‐seq) (Figures 3A and S3A). In the TCGA_HNC cohort, multivariable regression models (controlling for age and sex) revealed that a high *CHRNA5* level was related to an increase in the T and N stages, and worse OS/DFS, while a high *CHRNA7* level was related to a decreased T stage (Figure 3B). In the group with a high *CHRNA5* level (top half vs. bottom half), the patients with a high *CHRNA5* level alone had the worst DFS, followed by the patients with high *CHRNA5* and *CHRNA7* levels (Figure 3B). In the TCGA_OSCC cohort, high *CHRNA5* expression was associated with poorer DFS (Figure S3B). Separate survival regression analyses of *CHRNA3*, *CHRNA5*, and *CHRNA7*, adjusted for age and sex, yielded results largely consistent with a combined model including all three genes in both the TCGA_HNC and TCGA_OSCC cohorts (Figure S4).

Regarding the epigenetic data, high *CHRNA5* expression was associated with a lower hyper/hypo‐methylation ratio of genes (0.18 and 0.02) and a higher over/under‐expressed miRNA ratio (9.55 and 31) when compared with high *CHRNA3* and *CHRNA7* expression in both the TCGA_HNC and TCGA_OSCC cohorts (Figure 3C,D). Figure S3C,D, these findings suggested that *CHRNA5* facilitated tumor progression via hypomethylation and miRNA production and had a greater prognostic impact on HNC patients than *CHRNA3* and *CHRNA7*.

Further GSEA (the first quarter vs. the last quarter) revealed that high *CHRNA3* expression was related to an increase in ion channel activities and cell adhesion/junction, and a decrease in antigen presentation and ATP synthesis (Figure 3E). High *CHRNA5* expression was related to a decrease in cell adhesion/junction, immune responses, and major histocompatibility complex (MHC) protein binding (Figure 3F). High *CHRNA7* expression was related to an increase in cell adhesion/junction, epithelial‐mesenchymal transition (EMT), immune responses, and MHC protein binding, and a decrease in ATP synthesis and oxidoreductase activities (Figure 3G). GSEA results were similar in both the TCGA_HNC and TCGA_OSCC cohorts (Figure S3E–G). These findings suggested that the differential expression of *CHRNA3, 5*, and *7* might influence HNC to exhibit different biological behaviors.

### 3.6 High Levels of *CHRNA3* and *CHRNA5* and Low Levels of *CHRNA7* Were Related to Increased Resistance to the Majority of Inhibitors in DepMap HNC Cell Lines

**FIGURE 4:**
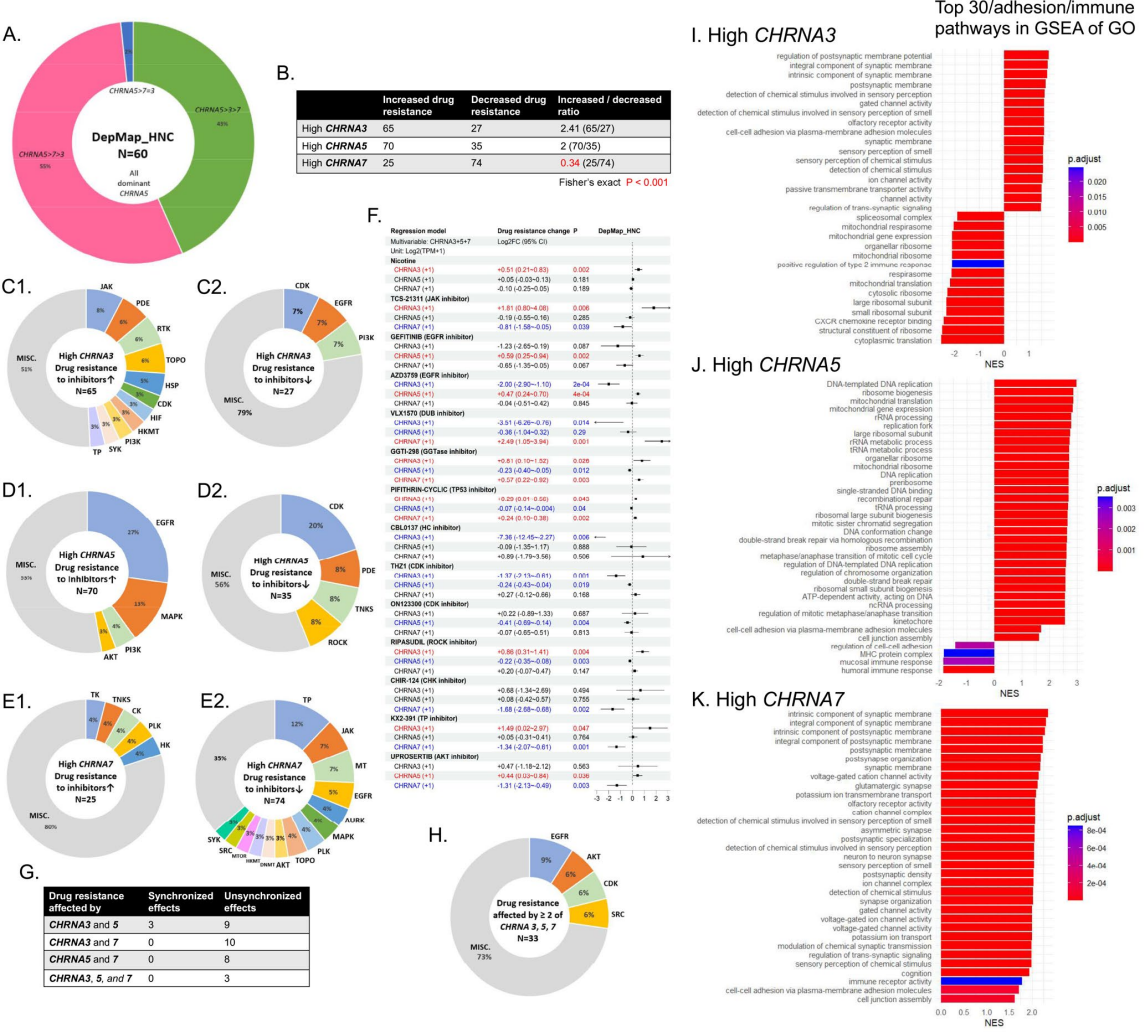
Influences of *CHRNA3*, *CHRNA5*, and *CHRNA7* expression in DepMap HNC cell lines. (A) The expression patterns of *CHRNA3*, *CHRNA5*, and *CHRNA7* in DepMap HNC cell lines. (B) The number of inhibitors whose drug sensitivity was affected by the expression of *CHRNA3*, *CHRNA5*, and *CHRNA7*. (C1–C2, D1–D2, E1–E2) The types of inhibitors whose drug sensitivity was affected by the expression of *CHRNA3*, *CHRNA5*, and *CHRNA7*, respectively. (F) The resistance changes to nicotine and inhibitors affected by the expression of *CHRNA3*, *CHRNA5*, and *CHRNA7*. (G–H) The number and types of inhibitors whose drug sensitivity was affected by at least two of *CHRNA3*, *CHRNA5*, and *CHRNA7*. (I–K) The enriched pathways in GSEA:GO for high *CHRNA3*, *CHRNA5*, and *CHRNA7* expression, respectively. HNC, head and neck cancer; FC, Fold change; TPM, transcripts per million; CI, confidence interval; GSEA, gene set enrichment analysis; GO, Gene ontology; JAK, Janus kinase; PDE, phosphodiesterase; RTK, receptor tyrosine kinase; TOPO, topoisomerase; HSP, heat shock protein; CDK, cyclin‐dependent kinase; HIF, hypoxia‐inducible factor; HKMT, histone lysine methyltransferase. PI3K, phosphoinositide 3‐kinase. SYK, spleen tyrosine kinase; TP, tubulin polymerization; EGFR, epidermal growth factor receptor; MAPK, mitogen‐activated protein kinase; AKT, protein kinase B; TNKS, Tankyrase ROCK, Rho kinase; TK, Tyrosine kinase; CK, casein kinase; PLK, Polo‐like kinase; HK, hexokinase; MT, microtubule; AURK, aurora kinase; DNMT, DNA methyltransferase; MTOR, mammalian target of rapamycin.

Sixty HNC cell lines were recognized in the DepMap database, in which all of them were *CHRNA5‐*dominant (RNA‐seq), including the SCC‐4 cell line (Figure 4A). The drug sensitivity data (log2 fold change method) of nicotine and 3117 inhibitors were then retrieved for these HNC cell lines. Multivariable regression models combining the expression of *CHRNA3, CHRNA5, and CHRNA7* revealed that high *CHRNA7* expression was related to the least number of drugs showing increased resistance and the greatest number of drugs showing decreased resistance (Figure 4B). High *CHRNA3* expression was associated with increased resistance mainly to Janus kinase (JAK) inhibitors and decreased resistance to the inhibitors of cyclin‐dependent kinase (CDK), epidermal growth factor receptor (EGFR), and phosphoinositide 3‐kinase (PI3K) (Figure 4C). High *CHRNA5* expression was associated with increased resistance to the inhibitors of EGFR, mitogen‐activated protein kinase (MAPK), PI3K, and protein kinase B (AKT), and decreased resistance primarily to CDK inhibitors (Figure 4D). High *CHRNA7* expression was associated with increased resistance to but not limited to tyrosine kinase (TK) inhibitors and decreased resistance chiefly to the inhibitors of tubulin polymerization (TP), JAK, and microtubule (MT) (Figure 4E). More detailed effects of *CHRNA3*, *CHRNA5*, and *CHRNA7* on drug resistance are shown in Figure 4F. The effects of nicotine were only related to *CHRNA3* levels, which were compatible with our qRT‐PCR results. For inhibitors whose drug sensitivities were affected by at least two of *CHRNA3*, *CHRNA5*, or *CHRNA7*, primarily the inhibitors of EGFR, AKT, CDK, and SRC, the effects of *CHRNA3*, *CHRNA5*, or *CHRNA7* were almost unsynchronized (Figure 4G,H). These findings suggested that high *CHRNA3* or *CHRNA*5 levels with low *CHRNA7* levels might indicate increased resistance to the majority of inhibitors. EGFR inhibitors seemed to be effective in treating high *CHRNA3* or *CHRNA7‐*expressed HNC cells but not high *CHRNA5*‐expressed HNC cells, which could be treated effectively by CDK inhibitors.

Further GSEA (the first quarter vs. the last quarter) revealed that high *CHRNA3* expression was related to an increase in ion channel activities and a decrease in immune responses and mitochondrial respirasome activities (Figure 4I). High *CHRNA5* expression was related to an increase in DNA replication and double‐strand break repair (which might increase drug resistance), a decrease in MHC complex presentation and immune responses, and an increase/decrease in cell adhesion/junction (Figure 4J). High *CHRNA7* expression was related to an increase in ion channel activities, immune receptor activities, and cell adhesion/junction (Figure 4K). These findings were generally compatible with our TCGA analyses.

### 3.7 The Presence and Expression Levels of *CHRNA3* , *CHRNA5* , and *CHRNA7* Affected Cell Adhesion and Immune Responses in HNC Cells (Puram 2017)

**FIGURE 5:**
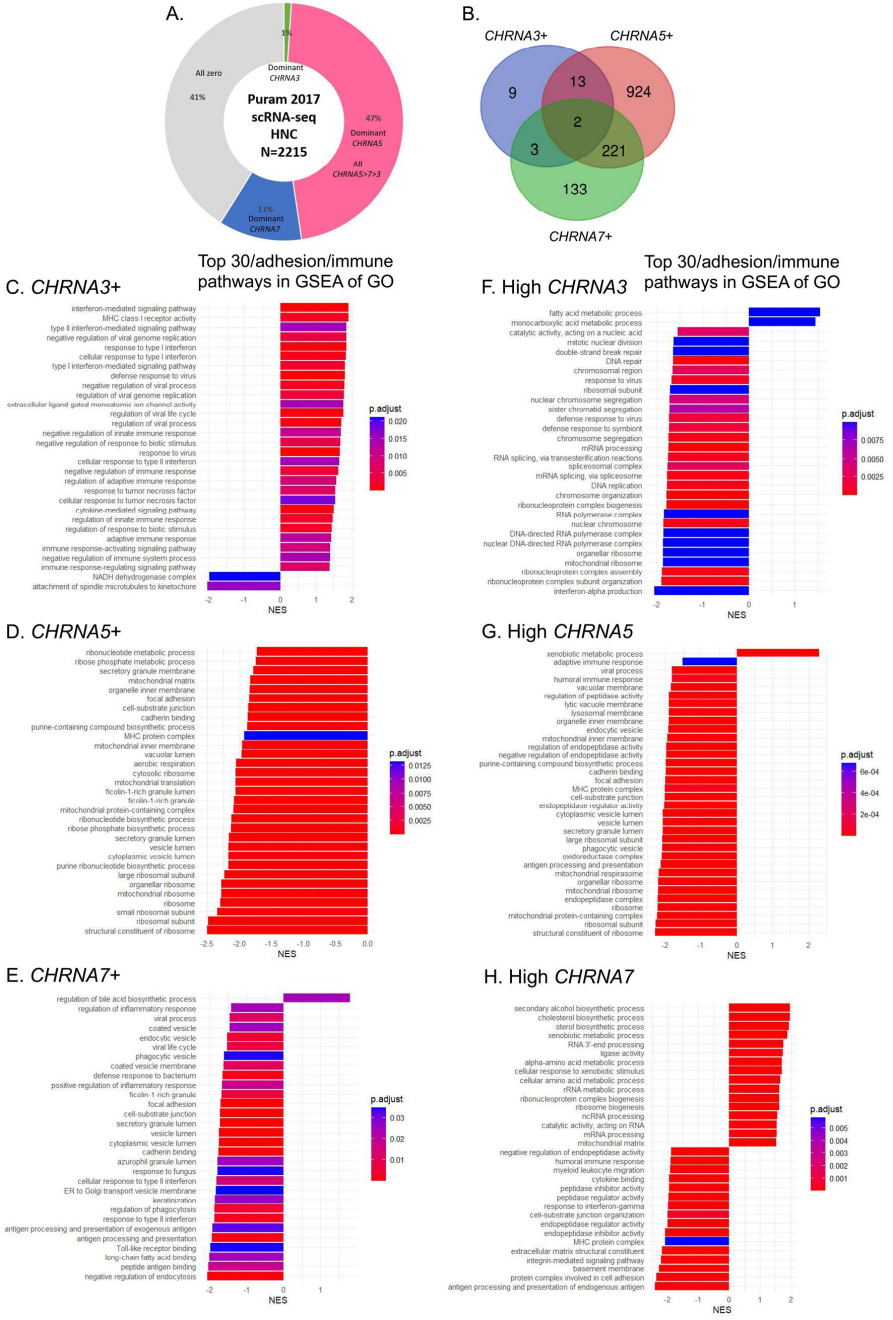
Influences of *CHRNA3*, *CHRNA5*, and *CHRNA7* expression in Puram 2017 (scRNA‐seq). (A, B) The expression patterns of *CHRNA3*, *CHRNA5*, and *CHRNA7* in the tumor cells of HNC patients. (C–E) The enriched pathways in GSEA:GO for the presence of *CHRNA3*, *CHRNA5*, and *CHRNA7*, respectively. (F–H) The enriched pathways in GSEA:GO for high *CHRNA3*, *CHRNA5*, and *CHRNA7* expression, respectively. GSEA, gene set enrichment analysis; GO, Gene ontology.

The transcriptomes (scRNA‐seq) of 2215 tumor cells from 18 HNC patients (Puram 2017) were analyzed, in which *CHRNA5*‐dominant cells were the most (47%), followed by the cells lacking expression of *CHRNA3*, *CHRNA5*, and *CHRNA7* (41%) (Figure 5A). The number of cells that expressed exclusively one of the three genes was 9, 924, and 133 for *CHRNA3*, *CHRNA5*, and *CHRNA7*, respectively (Figure 5B). These cells were compared to the cells without expression of all three genes by GSEA (Figure 5C–E). Another GSEA (the first quarter vs. the last quarter) was performed for *CHRNA3*+, *CHRNA5*+, and *CHRNA7*+ cells, respectively (Figure 5F–H). *CHRNA3* presentation was related to an increase in ion channel activities and immune responses (Figure 5C). *CHRNA5* presentation was related to a decrease in cell adhesion/junction, metabolic processes, MHC complex presentation, and immune responses (Figure 5D). *CHRNA7* presentation was related to a decrease in cell adhesion/junction and antigen presentation (Figure 5E). High *CHRNA3* expression was related to an increase in metabolic processes and a decrease in DNA replication and repair (Figure 5F). High *CHRNA5* expression was related to an increased metabolic process and a decrease in cell adhesion/junction, MHC complex presentation, and immune responses (Figure 5G). High *CHRNA7* expression was related to increased metabolic processes and a decrease in cell adhesion/junction, immune responses, and antigen presentation (Figure 5H). Both the presence and high expression of *CHRNA5* and *CHRNA7* were related to a decrease in cell adhesion and immune responses.

### 3.8 The Different Biological Effects of *CHRNA3* , *CHRNA5* , and *CHRNA7* Were Enriched in the STRING PPI Networks

**FIGURE 6:**
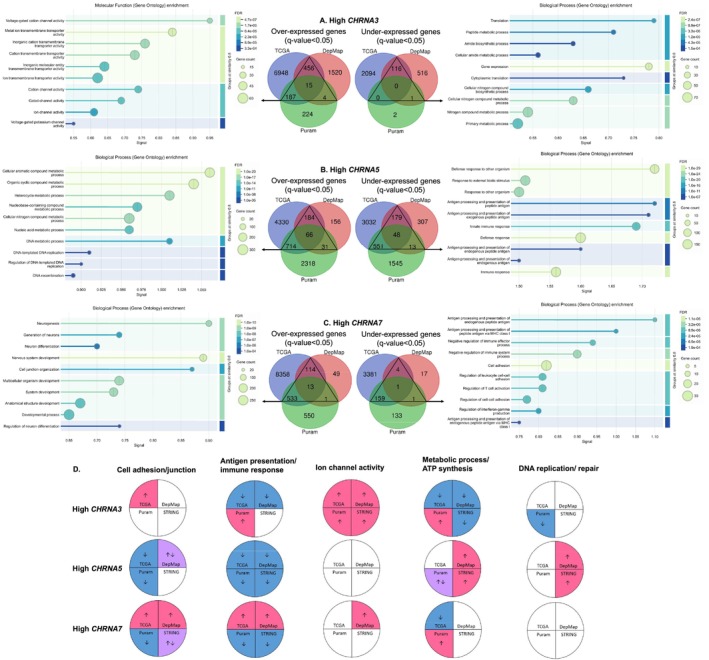
Influences of *CHRNA3*, *CHRNA5*, and *CHRNA7* expression in the STRING PPI networks. (A–C) The enriched pathways of GO for the overlapping DEGs from at least two of three databases, associated with high *CHRNA3*, *CHRNA5*, and *CHRNA7* expression, respectively. (D) Comparisons of the biological effects of *CHRNA3*, *CHRNA5*, and *CHRNA7* as revealed by different databases. DEGs, differentially expressed genes.

The overlapping DEGs from at least two among three databases (TCGA, DepMap, and Puram 2017) associated with high *CHRNA3*, *CHRNA5*, or *CHRNA7* expression were subjected to STRING PPI analyses, respectively. High *CHRNA3* expression was related to increased ion channel activities and decreased metabolic processes (Figure 6A). High *CHRNA5* expression was related to increased metabolic processes and decreased antigen presentation/immune responses (Figure 6B). High *CHRNA7* expression was related to an increase in cell junction and differentiation, and a decrease in cell adhesion, antigen presentation, and immune responses (Figure 6C). These findings suggested that the core biological effects of *CHRNA3*, *CHRNA5*, and *CHRNA7* were different in HNC.

The biological effects of *CHRNA3*, *CHRNA5*, and *CHRNA7*, as revealed by four different databases, are compared in Figure 6D. The results for *CHRNA5* were generally comparable, while discrepancies were observed between the results from different databases for *CHRNA3* and *CHRNA7*, mainly in terms of cell adhesion, immune responses, and metabolic processes. High *CHRNA3* for increased ion channel activities and high *CHRNA5* for decreased immune responses were the most consistent outcomes across all databases. These findings indicated that the effects of *CHRNA5* might be strong enough to ensure consistency, while the effects of *CHRNA3* and *CHRNA7* might be more prone to being influenced by other factors.

## 4 Discussion

In this study, we explored the effects of *CHRNA3*, *CHRNA5*, and *CHRNA7* in OSCC using cell‐based experiments and pathological IHC, and further investigated their biological functions in HNC through bioinformatics analyses. The expression of *CHRNA3*, *CHRNA5*, *CHRNA7*, along with epithelial and mesenchymal markers mutually influenced each other. Their protein expression in tumor infiltration was associated with deteriorated invasion patterns. *CHRNA3* maintained ion channel activity (bioinformatics analyses), decreased epithelial features, and increased mesenchymal features, thereby supporting the EMT process. *CHRNA5* promoted DNA replication, reduced cell adhesion, suppressed antigen presentation, and induced hypomethylation and miRNA overexpression (bioinformatics analyses), and also attenuated epithelial features and enhanced mesenchymal traits, ultimately leading to a disseminated tumor pattern, a worse clinical stage, and a poor prognosis. *CHRNA7* promoted cell differentiation and exhibited variable effects on cell adhesion and antigen presentation (bioinformatics analyses), while also enhancing both epithelial and mesenchymal characteristics, thus facilitating the maintenance of a hybrid EMT state. Analysis of the DepMap database revealed that high levels of *CHRNA3* and *CHRNA5* and low levels of *CHRNA7* were related to increased resistance to the majority of inhibitors. EGFR inhibitors were effective in treating high *CHRNA3‐* or *CHRNA7*‐expressing HNC, while CDK inhibitors were effective in treating high *CHRNA5*‐expressing HNC. Accordingly, the proposed contributions of *CHRNA3*, *CHRNA5*, and *CHRNA*7 to the EMT process are illustrated in Figure S5.

Docetaxel (a microtubule inhibitor) and Cetuximab (an EGFR inhibitor) are commonly used in chemotherapy regimens to treat HNC today [33]. According to our DepMap analysis, these two drugs should be effective in treating HNC exhibiting high *CHRNA3* or *CHRNA7* expression, but not those with high *CHRNA5* expression. This is consistent with our TCGA_HNC findings in which the patients with high *CHRNA5* expression alone had a worse DFS than the patients with high *CHRNA3*, *CHRNA5*, and *CHRNA7* expression. Our DepMap findings also suggested that CDK inhibitors seemed to be beneficial in treating HNC exhibiting high *CHRNA5* expression. The Food and Drug Administration (FDA) has approved several CDK inhibitors for treating metastatic hormone receptor‐positive breast cancer, but not yet for HNC [34]. CDK inhibitors interfere with the cell cycle, leading to decreased DNA replication, which might counteract the DNA replication‐promoting effects of high *CHRNA5* expression observed in our analysis. Ongoing clinical trials are investigating the use of CDK inhibitors for treating HNC patients [35].

Cancer cells have been found to be inclined to maintain a hybrid EMT state, with strong capacities for proliferation, invasion, and metastasis [36]. To achieve this, cancer cells of epithelial origin (such as HNC) are expected to keep increasing mesenchymal features and modifying epithelial characteristics. This is consistent with our findings, where *CHRNA3*, *CHRNA5*, and *CHRNA*7 all increased mesenchymal features, while *CHRNA3* and *CHRNA5*, as well as *CHRNA7* had counteracting effects on epithelial characteristics. In addition, a hybrid EMT state is also characterized by decreased, but not completely lost, cell adhesion [36]. Our analysis suggested that *CHRNA3* was related to increased or unchanged cell adhesion, *CHRNA5* was primarily related to decreased cell adhesion, and *CHRNA7* was related to both increased and decreased cell adhesion. This is consistent with our IHC findings, where tumor infiltrations had higher *CHRNA5* levels than tumor islands, which was not the case for *CHRNA3* and *CHRNA7*. Although the gene expression and prognostic impacts of *CHRNA5* are usually stronger than those of *CHRNA3* and *CHRNA7*, all three appear to be involved in the establishment of hybrid EMT states. Previous studies revealed that *CHRNA3* knockout mice exhibited cell–cell detachment, while *CHRNA7* knockout mice featured decreased extracellular matrix proteins in the epidermis [37, 38]. Silencing *CHRNA5* was found to exert opposite effects compared to silencing *CHRNA7* on cell migration in lung cancer cells [39]. *CHRNA5* and *CHRNA7* were found to be upregulated in lung cancer, with *CHRNA5* mediating nicotine‐ and chronic stress–induced tumor progression, promoting cancer cell proliferation, migration, invasion, stemness, EMT, radio‐ and cisplatin resistance, and immune evasion, and *CHRNA7* mediating nicotine‐induced PD‐L1 expression via the STAT3 pathway, collectively contributing to tumor progression, immune evasion, and poor patient survival [40, 41, 42, 43, 44]. In colorectal cancer, high *CHRNA3* expression was found to be associated with poor prognosis and to exhibit a strong physical interaction with *CHRNA5* [45]. In laryngeal and hypopharyngeal cancers, nAChR subunit *α*5 expression was found to increase with tumor progression [46]. In OSCC, *CHRNA5* upregulation was found to drive radioresistance through E2F pathway activation, whereas *α*7‐nAChR was found to promote nicotine‐induced malignancy, enhance cell survival, and confer cisplatin resistance as well as Bcl‐2–mediated anti‐apoptotic signaling, together contributing to tumor progression and therapeutic resistance [47, 48, 49]. In HNC, *CHRNA5* was found to mediate nicotine‐induced proliferation, migration, and invasion by regulating CES1 via the MEK/ERK pathway, contributing to tumor recurrence and metastasis [50]. A database analysis revealed that among nAChR subunits, *CHRNA5* was the most frequently upregulated in cancer tissues compared with normal tissues, including bladder, cervical, breast, uterine, lung, stomach, esophageal, colorectal, skin, brain, thyroid cancers, and others [5]. These findings supported our results in HNC.

Regarding immune responses, a former study illustrated that lung metastatic mammary cancer cells in mice with clustered patterns had increased MHC I expression, while those with disseminated patterns had decreased MHC I expression, indicating the relationships between tumor dissemination and immune escape [51]. Previous studies have also acknowledged that cancer hybrid EMT states interact with immune responses and promote immune evasion [52, 53, 54]. In addition, it has been noticed that nicotine and nAChRs are able to result in abnormal miRNA expression to downregulate immune reactions [55]. In our study, decreased cell adhesion was generally associated with decreased antigen presentation, while increased cell adhesion was typically associated with increased antigen presentation, especially for *CHRNA5* and *CHRNA7*. High *CHRNA5* was also associated with miRNA overexpression. These were compatible with previous findings.

Numerous nAChR downstream signaling pathways, including the Ras–Raf, Ca^2+^‐PKC‐Raf, PI3K‐Akt, and JAK–STAT pathways, have been reported to facilitate cancer proliferation [5, 6]. Different nAChR subunits appeared to influence the choice of pathway, in which subunit *α*3 was related to an activated PKC pathway in keratinocytes, subunit *α*5 was related to an activated JAK–STAT pathway in lung cancer, and subunit *α*7 was related to an activated PI3K‐Akt pathway in OSCC [5, 6, 40, 47, 56, 57]. These findings supported our results that *CHRNA3*, *CHRNA5*, and *CHRNA7* exerted different biological effects in HNC. Nevertheless, in our analyses, *CHRNA3* and *CHRNA7* expression influenced resistance to JAK inhibitors, while *CHRNA5* expression had little effect. *CHRNA3* and *CHRNA5* expression influenced resistance to PI3K inhibitors, while *CHRNA7* expression had little effect. These findings suggested that *CHRNA3*, *CHRNA5*, and *CHRNA7* might collaborate to modulate the biological behaviors of HNC.

As of 2022, the International Agency for Research on Cancer (IARC) has classified a total of 83 substances in unburned tobacco and tobacco smoke as carcinogens, including major groups such as N‐nitrosamines (TSNAs), polycyclic aromatic hydrocarbons (PAHs), aldehydes, volatile hydrocarbons, and aromatic amines [58]. Although nicotine is not classified as a carcinogen, it is the main addictive component of tobacco and promotes the development of cancers such as lung, head and neck, pancreatic, gastric, colon, breast, and bladder by inducing genotoxic DNA damage, activating nAChR signaling to enhance proliferation and metastasis, and suppressing anti‐tumor immunity [5, 6, 59]. Nicotine increases the protein expression of nAChRs and their subunits mainly through posttranscriptional pharmacological chaperoning, which enhances receptor maturation and assembly while reducing degradation [60]. However, nicotine generally has little effect on nAChR subunit mRNA expression, although *CHRNA3* transcripts are significantly reduced in smokers' lung tissues, consistent with our qRT‐PCR findings [60, 61]. Smoking cessation is the most effective strategy to prevent lung and head and neck cancers, reducing lung cancer incidence by up to 77% and HNC risk by about 60% in long‐term abstinent individuals compared with current smokers [62, 63]. Regarding the treatment of smoking‐induced cancers, selective nAChR antagonists may hold potential by precisely targeting overexpressed nAChR subunits in cancer cells and minimizing adverse effects on healthy tissues, with *α*7‐nAChR antagonists having been shown to inhibit lung cancer [5]. According to our results, *α*5‐nAChR antagonists, which are not currently available, might represent a superior option to *α*7‐nAChR antagonists for cancer treatment and therefore warrant further investigation.

The main limitation of our study was that in vivo experiments were not performed to explore the biological influence of nAChR subunits on OSCC. Another limitation was that only one cell line was used in our in vitro experiments. Although bioinformatic analyses across multiple databases in our study provided significant insights into the molecular and prognostic influence of different nAChR subunits in HNC, further functional validation experiments are required. In addition to overexpression, qRT‐PCR, and RNA‐seq in OSCC cells, and IHC in OSCC pathological specimens, our findings need to be further validated by multiple experimental approaches such as protein functional assays, Western blotting, and gene silencing in different HNC cancer cell lines as well as in vivo animal models. Inhibitors targeting different nAChR subunits should also be screened, with their antitumor efficacy and toxicity further evaluated in vivo. In summary, the differential expression of *CHRNA3, CHRNA5, and CHRNA7* exerted prognostic and biological impacts on OSCC/HNC. *CHRNA3* maintained the ion channel activities of nAChRs and frequently contributed to increased drug resistance. *CHRNA5* promoted DNA replication, decreased cell adhesion and antigen presentation, commonly contributed to increased drug resistance, and worsened survival. *CHRNA7* promoted differentiation, exhibited variable effects on cell adhesion and antigen presentation, and usually contributed to decreased drug resistance. The findings might shed light on future exploring potential therapeutic approaches for OSCC/HNC.

## Author Contributions

**Chi‐Maw Lin:** conceptualization, writing – original draft, funding acquisition, formal analysis, methodology, data curation. **Long‐Wei Lin:** methodology, visualization. **Tseng‐Cheng Chen:** validation, investigation, supervision. **Yi‐Ling Ye:** funding acquisition, investigation, validation, supervision. **Bor‐Luen Chiang:** writing – original draft, writing – review and editing, supervision, data curation.

## Ethics Statement

Approval of the research protocol by an Institutional Review Board: The Research Ethics Committee of the National Taiwan University Hospital approved this study (NTUH IRB‐201910107RINC).

## Consent

The authors have nothing to report.

## Conflicts of Interest

The authors declare no conflicts of interest.

## Supporting information


**Table S1:** Clinical characteristics of patients in our series and in the TCGA database.

**Figure S1:** Schematic representation of nAChRs and predicted protein structures of the *α*3, *α*5, and *α*7 subunits (AlphaFold Protein Structure Database). nAChR, nicotinic acetylcholine receptor; pLDDT, predicted local distance difference test.
**Figure S2:** The effects of nAChR α3, α5, and α7 subunit expression on survival in OSCC pathological specimens assessed by IHC, with each subunit analyzed separately and adjusted for age and sex. (A) nAChR α3 alone. (B) nAChR α5 alone. (C) nAChR α7 alone. nAChR, nicotinic acetylcholine receptor. OSCC, oral squamous cell carcinoma; IHC, immunohistochemistry; HR, hazard ratio; CI, confidence interval; OS, overall survival; DFS, disease‐free survival.
**Figure S3:** Influences of CHRNA3, CHRNA5, and CHRNA7 expression in the TCGA‐OSCC cohort. (A) Clinical characteristics of the TCGA‐OSCC cohort. (B) 1‐3, The effects of CHRNA3, CHRNA5, and CHRNA7 expression on pathological features and survival. (C) The effects of CHRNA3, CHRNA5, and CHRNA7 expression on DNA methylation. (D) The effects of CHRNA3, CHRNA5, and CHRNA7 expression on miRNA production. (E‐G) The enriched pathways in GSEA:GO for high CHRNA3, CHRNA5, and CHRNA7 expression, respectively. OSCC, oral squamous cell carcinoma; OR, odds ratio; HR, hazard ratio; CI, confidence interval; GSEA, gene set enrichment analysis; GO: gene ontology.
**Figure S4:** The effects of CHRNA3, CHRNA5, and CHRNA7 expression on survival in the TCGA_HNC (A1‐A3) and TCGA_OSCC (B1‐B3) cohorts, with each subunit analyzed individually and adjusted for age and sex. TCGA, The Cancer Genome Atlas. HNC, head and neck cancer; OSCC, oral squamous cell carcinoma; HR, hazard ratio; CI, confidence interval; OS, overall survival; DFS, disease‐free survival.
**Figure S5:** Proposed contributions of CHRNA3, CHRNA5, and CHRNA7 to the EMT process. EMT, epithelial mesenchymal transition; EGFR, epidermal growth factor receptor; CDK, cyclin‐dependent kinase. ⊣, effective suppression.

## Data Availability

The data that support the findings of this study are available on request from the corresponding author. The data are not publicly available due to privacy or ethical restrictions.
