# Supplementary material for: The Biological and Prognostic Implications of the Nicotinic Acetylcholine Receptor α3, α5, and α7 Subunits in Oral Squamous Cell Carcinoma
Source: Cancer Med. 2025 Nov 7;14(21):e71358. doi: 10.1002/cam4.71358 (PMC12593526; doi:10.1002/cam4.71358)
Supplement: Supplementary file 1 — Table S1: Clinical characteristics of patients in our series and in the TCGA database. [file CAM4-14-e71358-s002.docx]

| **Supplementary Table S1.** Clinical characteristics of patients in our series and in the TCGA database | | | |
| --- | --- | --- | --- |
|  | **Our series**  **IHC_OSCC** | **TCGA** | |
|  |  | **TCGA_HNC** | **TCGA_OSCC** |
| **Patient number** | 57 | 500 | 308 |
| **Age (mean (standard deviation)) (years)** | 59.9 (10.11) | 61.08 (11.92) | 61.8 (13.08) |
| **Male/Female (ratio of males)** | 52/5 (91.23%) | 367/133 (73.4%) | 208/100 (67.53%) |
| **T stage** | | | |
| T1, 2 | 44 (77.19%) | 177 (35.4%) | 124 (40.26%) |
| T3, 4 | 13 (22.81%) | 267 (53.4%) | 166 (53.9%) |
| Unknown | 0 (0%) | 56 (11.2%) | 18 (5.84%) |
| **N stage** | | | |
| N0 | 39 (68.42%) | 171 (34.2%) | 116 (37.66%) |
| N1, 2, 3 | 18 (31.58%) | 236 (47.2%) | 148 (48.05%) |
| Unknown | 0 (0%) | 93 (18.6%) | 44 (14.29%) |
| **Smoking** | | | |
| Yes | 31 (54.39%) | 307 (61.4%) | 167 (54.22%) |
| No or quit > 15 years | 26 (45.6%) | 183 (36.6%) | 135 (43.83%) |
| Unknown | 0 (0%) | 10 (2%) | 6 (1.95%) |
| **Alcohol** | | | |
| Yes | 18 (31.58%) | 280 (56%) | 162 (52.6%) |
| No or currently quit | 39 (68.42%) | 209 (41.8%) | 139 (45.13%) |
| Unknown | 0 (0%) | 11 (2.2%) | 7 (2.27%) |
| **Betel nut** | | | |
| Yes | 26 (45.61%) | 0 (0%) | 0 (0%) |
| No or quit > 15 years | 31 (54.39%) | 0 (0%) | 0 (0%) |
| Unknown | 0 (0%) | 500 (100%) | 308 (100%) |
| *IHC, immunohistochemistry; TCGA, The Cancer Genome Atlas; OSCC, oral squamous cell carcinoma; HNC, Head and neck cancer* | | | |
