# Supplementary material for: The Biological and Prognostic Implications of the Nicotinic Acetylcholine Receptor α3, α5, and α7 Subunits in Oral Squamous Cell Carcinoma
Source: Cancer Med. 2025 Nov 7;14(21):e71358. doi: 10.1002/cam4.71358 (PMC12593526; doi:10.1002/cam4.71358)
Supplement: Supplementary file 2 — Figure S1: Schematic representation of nAChRs and predicted protein structures of the α3, α5, and α7 subunits (AlphaFold Protein Structure Database). nAChR, nicotinic acetylcholine receptor; pLDDT, predicted local distance difference test. Figure S2: The effects of nAChR α3, α5, and α7 subunit expression on survival in OSCC pathological specimens assessed by IHC, with each subunit analyzed separately and adjusted for age and sex. (A) nAChR α3 alone. (B) nAChR α5 alone. (C) nAChR α7 alone. nAChR, nicotinic acetylcholine receptor. OSCC, oral squamous cell carcinoma; IHC, immunohistochemistry; HR, hazard ratio; CI, confidence interval; OS, overall survival; DFS, disease‐free survival. Figure S3: Influences of CHRNA3, CHRNA5, and CHRNA7 expression in the TCGA‐OSCC cohort. (A) Clinical characteristics of the TCGA‐OSCC cohort. (B) 1‐3, The effects of CHRNA3, CHRNA5, and CHRNA7 expression on pathological features and survival. (C) The effects of CHRNA3, CHRNA5, and CHRNA7 expression on DNA methylation. (D) The effects of CHRNA3, CHRNA5, and CHRNA7 expression on miRNA production. (E‐G) The enriched pathways in GSEA:GO for high CHRNA3, CHRNA5, and CHRNA7 expression, respectively. OSCC, oral squamous cell carcinoma; OR, odds ratio; HR, hazard ratio; CI, confidence interval; GSEA, gene set enrichment analysis; GO: gene ontology. Figure S4: The effects of CHRNA3, CHRNA5, and CHRNA7 expression on survival in the TCGA_HNC (A1‐A3) and TCGA_OSCC (B1‐B3) cohorts, with each subunit analyzed individually and adjusted for age and sex. TCGA, The Cancer Genome Atlas. HNC, head and neck cancer; OSCC, oral squamous cell carcinoma; HR, hazard ratio; CI, confidence interval; OS, overall survival; DFS, disease‐free survival. Figure S5: Proposed contributions of CHRNA3, CHRNA5, and CHRNA7 to the EMT process. EMT, epithelial mesenchymal transition; EGFR, epidermal growth factor receptor; CDK, cyclin‐dependent kinase. ⊣, effective suppression. [file CAM4-14-e71358-s001.pdf]

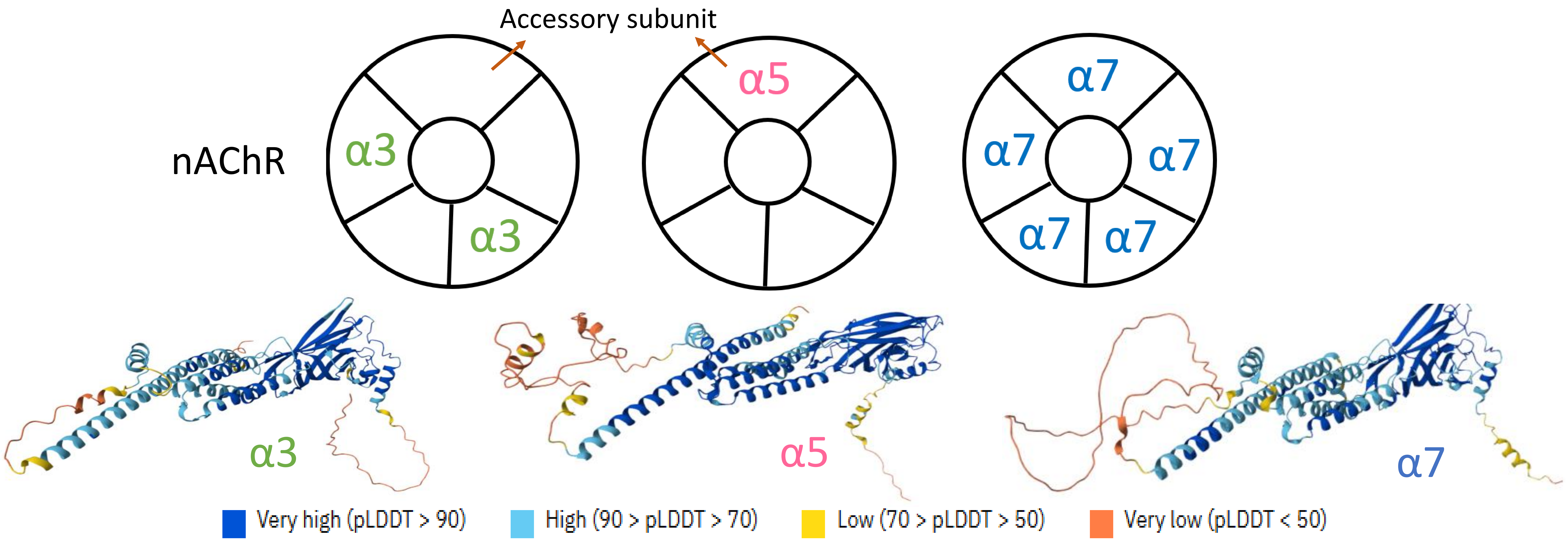

Supplementary Figure S1. Schematic representation of nAChRs and predicted protein structures of the α3, α5, and α7 subunits (AlphaFold Protein Structure Database). nAChR, nicotinic acetylcholine receptor. pLDDT, predicted local distance difference test.

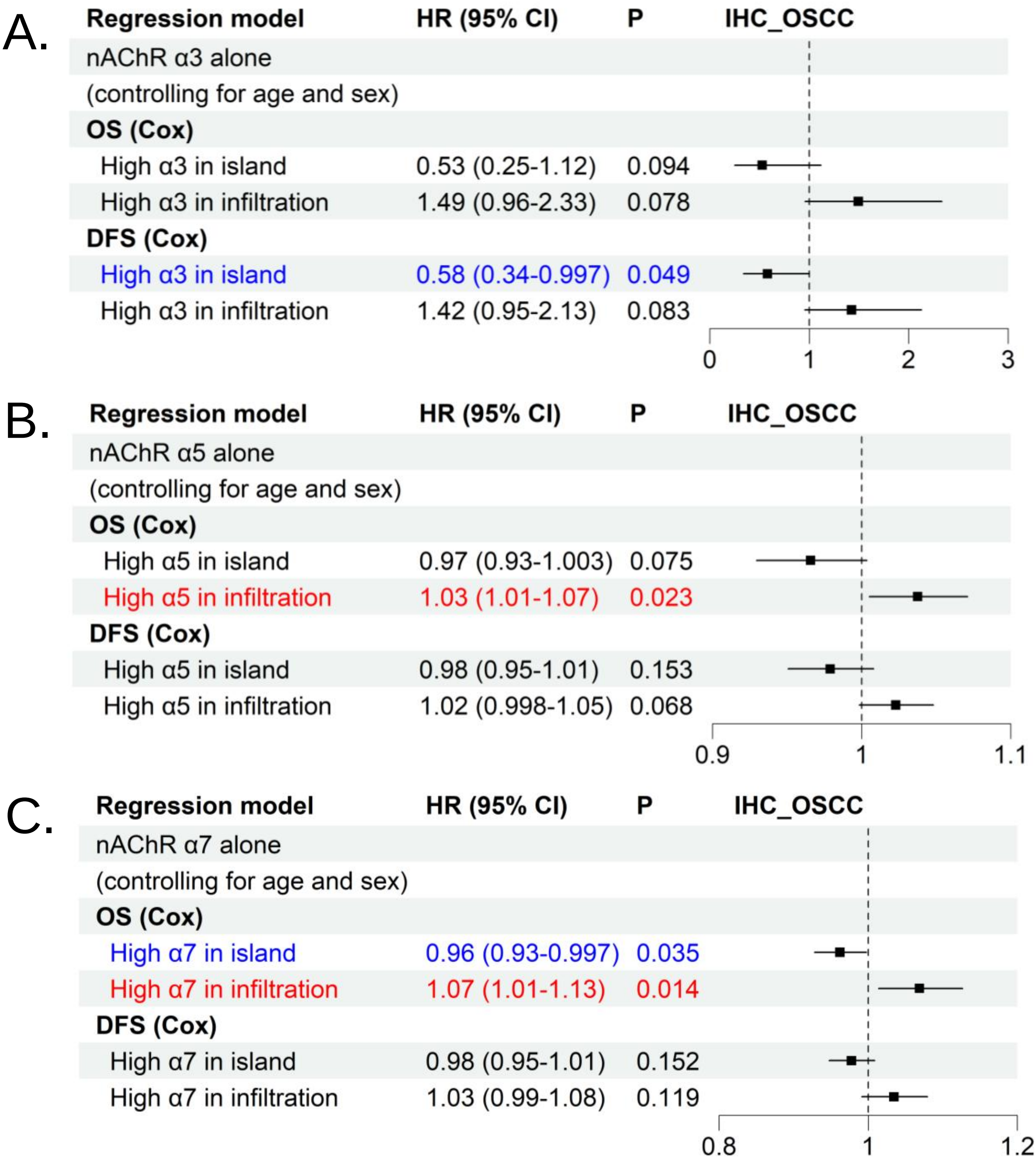

Supplementary Figure S2. The effects of nAChR α3, α5, and α7 subunit expression on survival in OSCC pathological specimens assessed by IHC, with each subunit analyzed separately and adjusted for age and sex. **A,** nAChR α3 alone. **B,** nAChR α5 alone. **C,** nAChR α7 alone. nAChR, nicotinic acetylcholine receptor. OSCC, oral squamous cell carcinoma. IHC, immunohistochemistry. HR, hazard ratio. CI, confidence interval. OS, overall survival, DFS, disease-free survival.

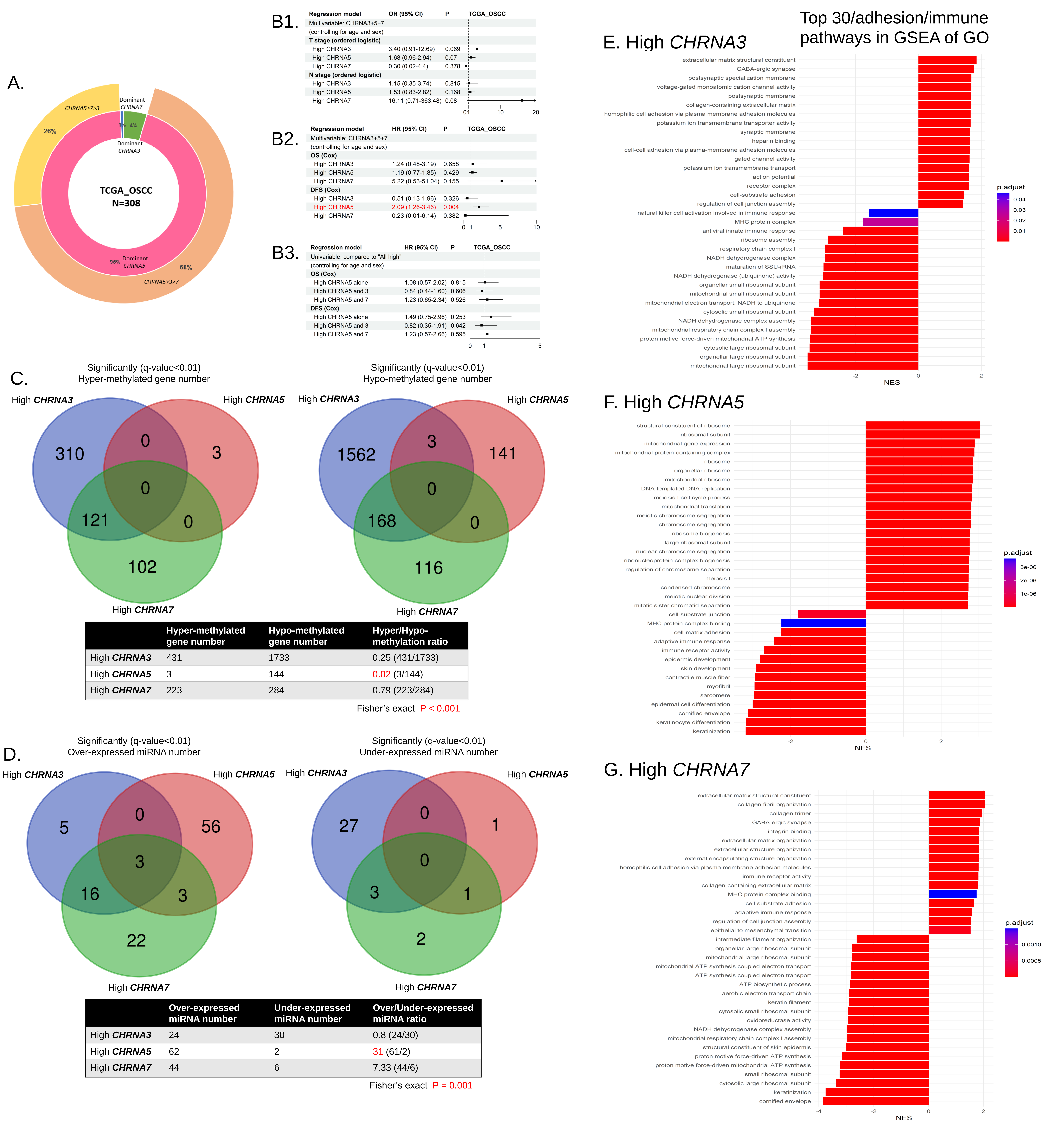

	Hyper-methylated gene number	Hypo-methylated gene number	Hyper/Hypo-methylation ratio
High *CHRNA3*	431	1733	0.25 (431/1733)
High *CHRNA5*	3	144	0.02 (3/144)
High *CHRNA7*	223	284	0.79 (223/284)

	Over-expressed miRNA number	Under-expressed miRNA number	Over/Under-expressed miRNA ratio
High *CHRNA3*	24	30	0.8 (24/30)
High *CHRNA5*	62	2	31 (61/2)
High *CHRNA7*	44	6	7.33 (44/6)

Supplementary Figure S3. Influences of *CHRNA3*, *CHRNA5*, and *CHRNA7* expression in the TCGA-OSCC cohort. A, Clinical characteristics of the TCGA-OSCC cohort. B1-3, The effects of *CHRNA3*, *CHRNA5*, and *CHRNA7* expression on pathological features and survival. C, The effects of *CHRNA3*, *CHRNA5*, and *CHRNA7* expression on DNA methylation. D, The effects of *CHRNA3*, *CHRNA5*, and *CHRNA7* expression on miRNA production. E-G, The enriched pathways in GSEA:GO for high *CHRNA3*, *CHRNA5*, and *CHRNA7* expression, respectively. OSCC, oral squamous cell carcinoma. OR, odds ratio. HR, hazard ratio. CI, confidence interval. GSEA, gene set enrichment analysis. GO: gene ontology.

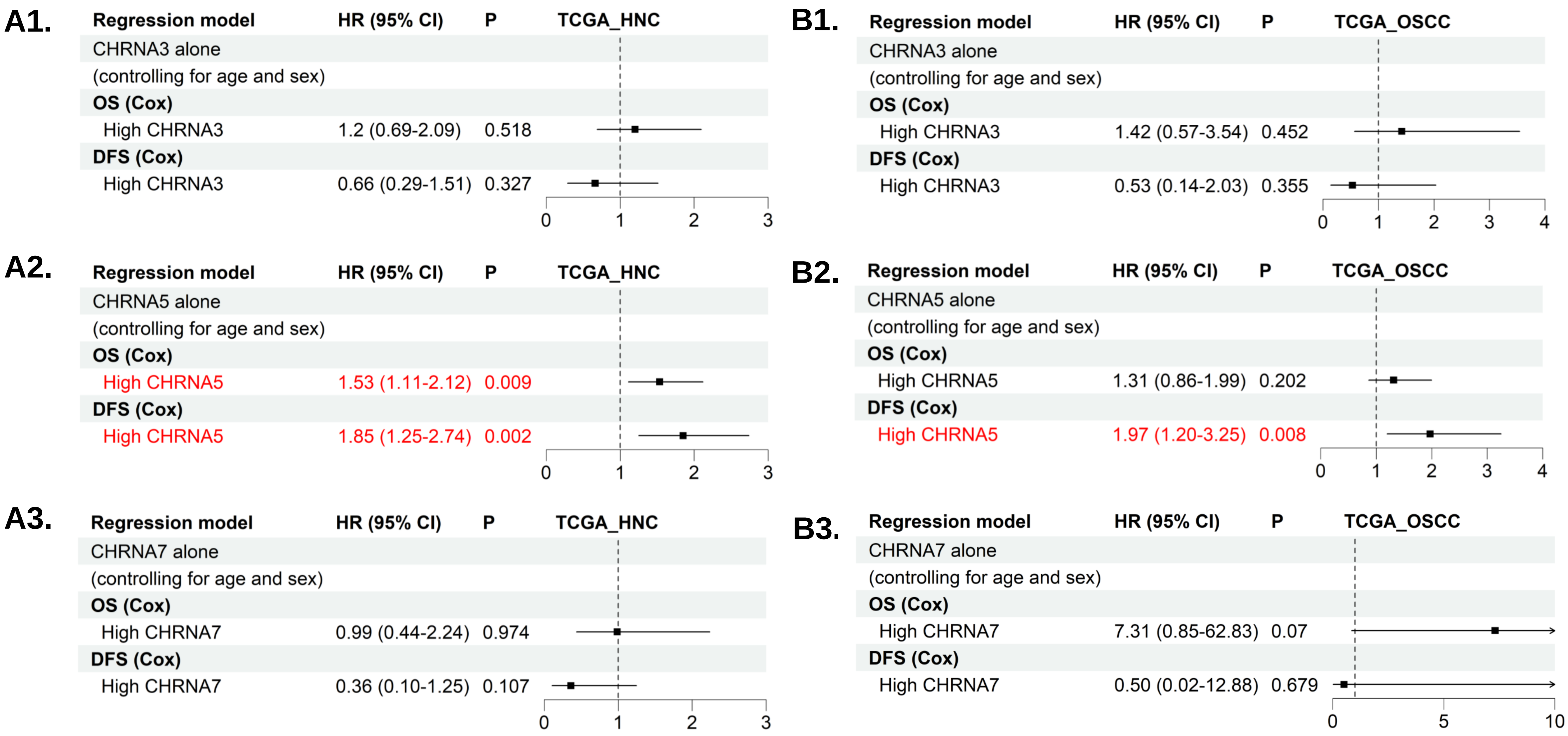

Supplementary Figure S4. The effects of *CHRNA3*, *CHRNA5*, and *CHRNA7* expression on survival in the TCGA_HNC (**A1-A3**) and TCGA_OSCC (**B1-B3**) cohorts, with each subunit analyzed individually and adjusted for age and sex. TCGA, The Cancer Genome Atlas. HNC, head and neck cancer. OSCC, oral squamous cell carcinoma. HR, hazard ratio. CI, confidence interval. OS, overall survival, DFS, disease-free survival.

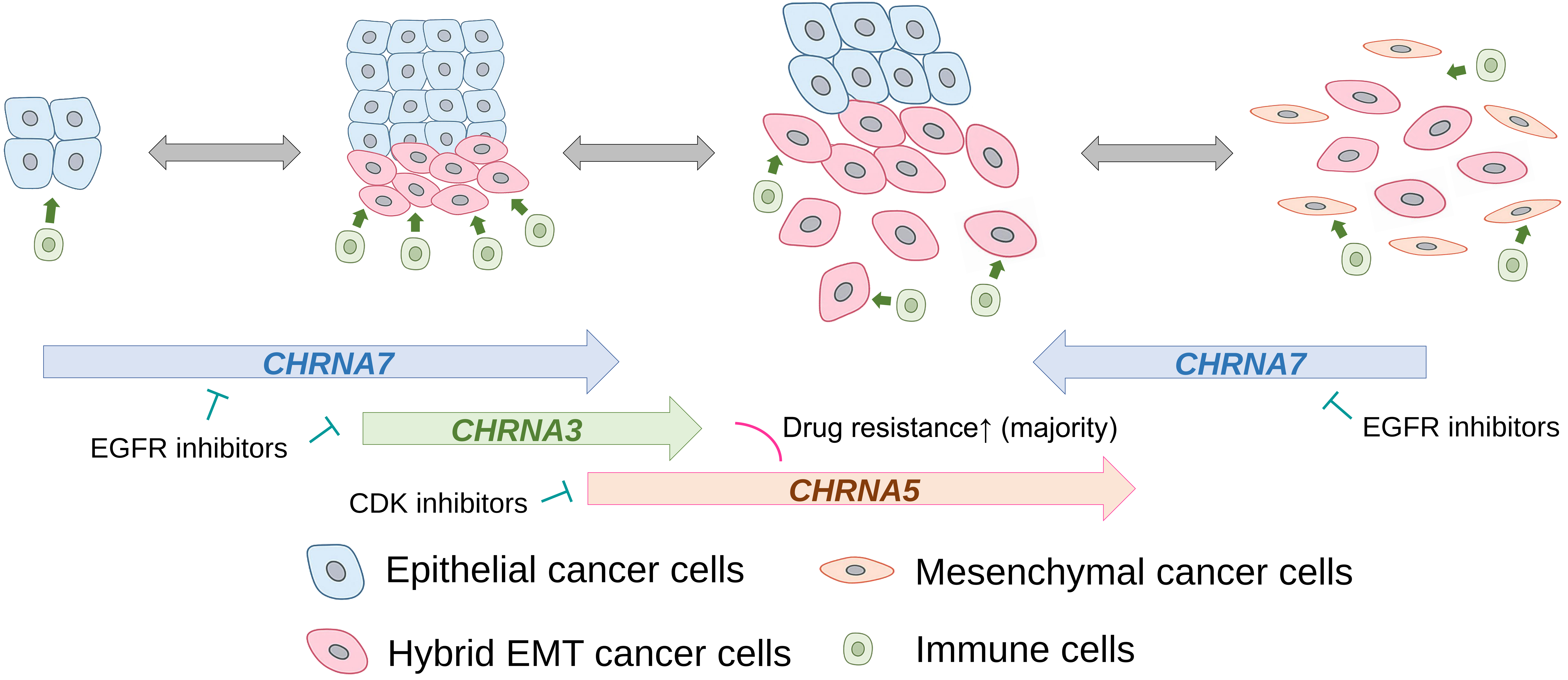

Supplementary Figure S5. proposed contributions of *CHRNA3*, *CHRNA5*, and *CHRNA7* to the EMT process. EMT, epithelial-mesenchymal transition. EGFR, epidermal growth factor receptor. CDK, cyclin-dependent kinase. ⊣, effective suppression.
